# Osteopontin promotes tumor growth and metastasis and GPX4-mediated anti-lipid peroxidation in triple-negative breast cancer by activating the PI3k/Akt/mTOR pathway

**DOI:** 10.1007/s00432-024-05658-w

**Published:** 2024-03-25

**Authors:** Man Guo, Mengyue Liu, Weihan Li, Cao Wang, Lu Zhang, Hao Zhang

**Affiliations:** 1 Department of Thyroid and Breast Surgery, Nanyang Central Hospital, No. 312 Gongnong Road, Wancheng District, Nanyang City, 473005 Henan Province China; 2 Xinxiang Medical University, Xinxiang City, 453003 Henan Province China

**Keywords:** Osteopontin, Tumor sphere formation, Angiogenesis, Triple-negative breast cancer, PI3K/AKT/mTOR pathway, GPX4, Lipid peroxidation

## Abstract

**Purpose:**

Triple-negative breast cancer (TNBC) features high aggressiveness, metastasis rate, drug resistance as well as poor prognosis. Osteopontin (OPN) is a key protein in the process of osteogenesis and has emerged as a new tumor marker in recent years.

**Methods:**

Cell viability was tested with the CCK-8 kit. Transwell and wound healing were adopted to test cell invasive and migratory abilities. Tumor sphere formation was detected by tumor sphere formation assay. Human umbilical vein endothelial cell (HUVEC) tube formation assay was used to measure the angiogenesis of tumor cells. Western blot was applied for the estimation of the expression of cancer stem cell markers, angiogenesis-, signaling pathway-related proteins as well as OPN. Bioinformatics tools predicted OPN expression in breast cancer tissues. The levels of oxidative stress-related markers were assessed with ELISA. Following the overexpression of OPN in MD-MB-436 cells and the addition of the PI3K/AKT/mTOR pathway inhibitor LY294002, the aforementioned functional experiments were implemented again to investigate the mechanism. Finally, in vivo experiments of tumor-bearing mice were performed for further verification.

**Results:**

The proliferative, invasive, migratory and tumor sphere formation capabilities as well as angiogenesis of TNBC cells were conspicuously increased in contrast to non-TNBC cell lines. OPN expression in TNBC tissues and cells was dramatically enhanced. OPN upregulation significantly elevated cell proliferative, invasive and migratory capabilities as well as tumor sphere formation and angiogenesis. The mechanism might be achieved by activating PI3K/AKT/mTOR signaling to regulate glutathione peroxidase 4 (GPX4)-mediated anti-lipid peroxidation.

**Conclusion:**

OPN promoted tumor sphere formation and angiogenesis in TNBC by activating the PI3K/AKT/mTOR pathway to regulate GPX4-mediated anti-lipid peroxidation levels.

## Introduction

Being a kind of breast cancer, triple-negative breast cancer (TNBC) features a lack of immunohistochemical expression of estrogen receptor, progesterone receptor as well as human epidermal growth factor receptor (Gelmon et al. 2012). TNBC has strong aggressiveness and rapid metastasis, along with poor prognosis. TNBC has the highest mortality among all kinds of breast cancer and has low 5-year survival rate (Cinkaya et al. 2016). Therefore, the investigation of the TNBC mechanism and possible targeted therapy has become a research hotspot.

Osteopontin (OPN), a calcium-binding phosphorylated protein associated with malignant transformation, plays an apparent role in promoting cell adhesion, chemotaxis and tumor metastasis in vivo (Icer and Gezmen-Karadag 2018). That is to say, OPN is a factor involved in the regulation of malignant behaviors of tumor cells and can impart influence on the advancement of tumors. Abundant OPN expression in blood indicates lower survival rate and higher recurrence rate of non-small cell lung cancer patients (Liu et al. 2015). OPN is highly expressed in colorectal cancer and may be a regulatory factor for colorectal cancer development (Amilca-Seba et al. 2021). At present, the influences of OPN on the pathogenesis and development of breast cancer and its molecular mechanism have become a research hotspot. In particular, it is reported that OPN is highly expressed and can be used as a predictive biomarker of anti-epidermal growth factor receptor therapy in TNBC (Anborgh et al. 2018). Previous literature has supported that OPN mRNA level is increased in HER2-positive and triple-negative/basal-like tumors (Ortiz-Martínez et al. 2014). These results indicate that OPN plays an important role in the development of TNBC. However, the specific impacts and molecular regulatory mechanism of OPN in the occurrence and development of TNBC have been scarcely studied.

It was discovered that PI3K/AKT/mTOR signaling is frequently dysregulated in human malignancies (Glaviano et al. 2023) and blocking the PI3K/AKT/mTOR signaling may represent a novel strategy for TNBC therapy (Costa et al. 2018; Khan et al. 2019). Meanwhile, GPX4 plays a master role in blocking ferroptosis by catalyzing the reduction of lipid peroxides (Liu et al. 2023), and targeting GPX4-mediated anti-lipid peroxidation is a promising therapeutic method for TNBC (Ding et al. 2021; Chen et al. 2023). Intriguingly, a recent study has revealed that mTOR has a close correlation with GPX4 (Liu et al. 2021). Moreover, OPN may induce malignant phenotype of cells by activating the PI3K/AKT/mTOR pathway, contributing to enhanced cell proliferative and invasive capabilities, metastasis, angiogenesis as well as undesirable treatment efficacy (Santos et al. 2022). However, the regulatory relationship between OPN and PI3K/AKT/mTOR signaling pathway in TNBC and their effects on sphere formation, angiogenesis and GPX4-mediated anti-lipid peroxidation have not been reported so far.

In this paper, we will discuss the effects of OPN on TNBC tumor sphere formation and vascular formation as well as its mechanism. Our paper may provide a theoretical basis for the exploration of TNBC pathogenesis and possible targeted therapy.

## Materials and methods

### Bioinformatics tools

The UALCAN database (https://ualcan.path.uab.edu/) predicted OPN expression in breast cancer tissues.

### Cell culture and treatment

Normal mammary epithelial cell line MCF-10A, breast cancer cell line MCF-7 cells and TNBC cell line MDA-MB-436 cells were provided by Cell Bank of the Chinese Scientific Academy and were cultivated in DMEM (GIBCO, Grand Island, USA) with 10% FBS (GIBCO, Grand Island, USA) and 1% penicillin and streptomycin (GIBCO, Grand Island, USA) in a humidified incubator at 37 °C with 5% CO_2_. The culture medium was changed once every 2–3 days when the cells grew. Cells were seeded in a six-well plate (1 × 10^5^ cells per well). Upon reaching 80% confluence, cells were transfected with overexpression plasmid using Dharmafect 1 transfection reagent (Dharmacon). OPN was cloned into pCMV3-C-his vector (OPN-OE, Sino Biological) and the empty vector was regarded as the negative control. After 48 h, RT-qPCR was utilized to test the transfection efficiency. Before cell transfection, cells were pretreated with LY294002 (10 µM, Selleck) for 30 min.

HUVECs were provided by Cell Bank of the Chinese Scientific Academy and propagated in endothelial ECM cell medium (GIBCO, Grand Island, USA) supplemented with 10% FBS (GIBCO, Grand Island, USA), 1% endothelial cell growth supplement (GIBCO, Grand Island, USA) and 1% penicillin and streptomycin (GIBCO, Grand Island, USA) in a humidified incubator at 37 °C with 5% CO_2_. The culture medium was changed once every 2–3 days when the cells grew.

### RT-qPCR

Total RNA was harvested by TRIzol reagent (Gibco, Grand Island, NY, USA) in light of the recommended protocol. cDNA was synthesized with 1 μg total RNA in a 20-μl reaction volume using a Fermentas^®^ First-Strand cDNA Synthesis kit (Thermo Fisher Scientific, Inc.). Reverse transcriptase reaction was operated with Brilliant II Fast SYBR green QPCR master mix (20 μl; Agilent Technologies, Santa Clara, CA, USA). RT-qPCR was operated utilizing SYBR Premix Ex Taq TM (Takara Bio, Inc., Otsu, Japan) in 7500 FAST Real-Time PCR System (Bio- Rad Co., USA). The reaction thermocycling conditions were as follows: 95 °C for 5 min, followed by 40 cycles of 94 °C for 15 s, 60 °C for 20 s and 72 °C for 40 s. The mRNA expression levels were valued by the 2^−ΔΔCt^ method and GAPDH was used to normalize the data (Livak and Schmittgen 2001). The results are expressed as the fold changes. The sequences were designed by Guangzhou RiboBio Co., Ltd. OPN forward, 5'-AGCAGCTTTACAACAAATACCCAG-3', and reverse, 5'-TACTTGGAAGGGTCTGTGGG-3'; GAPDH forward, 5′-AATGGGCAGCCGTTAGGAAA-3′, and reverse, 5′-GCGCCCAATACGACCAAATC-3′.

### CCK8

Cell viability was assayed employing a CCK8 Kit (Beyotime Institute of Biotechnology) in light of the standard protocol. TNBC cells (1 × 10^3^/mL) were seeded into 96-well plates in 100 μL cell suspension/well. After 24, 48 and 72 h, CCK-8 solution (10 μL) was added to the cells for 1 h. The OD value was assessed at 450 nm.

### Wound healing assay

Cells (2 × 10^5^cells/well) were inoculated into six-well plates. When cells reached 90% confluence, a 200-µl plastic tip was used to make a straight line in the monolayer and the plates were rinsed with PBS. The cells were observed under an inverted microscope equipped with a camera (Leica DMI 4000) at 25 °C and this time was designated as 0 h. Afterwards, cells were maintained in an incubator. The migratory cells were evaluated applying Image J (v 1.5.1, National Institutes of Health, USA).

### Transwell assay

Cells were suspended in serum-free medium and then seeded into the upper chamber of transwell inserts coated with Matrigel™ (50 mg/l; 8-µm pore size; Corning, Inc.). The upper chambers were pre-coated with Matrigel (1 mg/ml), while the lower chambers were incubated with a medium carrying 10% FBS. The chambers were adopted for cell cultivation. After incubation for 24 h, the cells were subsequently exposed to 4% polyoxymethylene for fixation for 15 min as well as 1% crystal violet for staining for 30 min (all at room temperature). The inside of the membrane was gently wiped with a cotton swab to remove any cells that had not migrated. Cells were visualized employing a light microscope (magnification 200, Leica DM 4000).

### Tumor sphere formation assay

The cells were inoculated into six-well plates with 1000 cells per well after the corresponding treatment. After cultivation for 14 days, tumor spheres were formed. The tumor spheres were observed and recorded under an optical microscope (DM4M, Leica, Solms, Germany) at a magnification of 200 × .

### Western blot

The lysis of cells was carried out employing RIPA buffer (Shanghai Ruji Biotechnology Development Co., Ltd.). Subsequently, lysates were centrifuged, the supernatant was recovered and protein was quantified by a BCA kit (Beyotime). Then, 30 μg of proteins were loaded on a 10% SDS gel, resolved using SDS-PAGE and transferred to PVDF membranes (Merck Millipore) which were impeded by 5% milk in PBS–Tween 20. Then PVDF membranes were incubated with the corresponding primary antibodies: anti-CD44 (1:800, ab243894, Abcam), anti-CD24 (1:1000, ab179821, Abcam), anti-ALDH1 (1:1000, ab52492, Abcam), anti-VWF (1:1000, ab6994, Abcam), anti-CD31 (1:1000, ab9498, Abcam), anti-OPN (1:1000, ab228748, Abcam), anti-mTOR (1:1000, ab134903, Abcam), anti-p-PI3K (1:1000, ab278545 Abcam), anti-PI3K (1:1000, ab154598, Abcam), anti-p-AKT (1:800, ab38449, Abcam), anti-AKT (1:1000, ab8805, Abcam), anti-GPX4 (1:1000, ab125066, Abcam), anti-LC3B (1:2000, ab192890, Abcam), anti-Beclin1 (1:2000, ab207612, Abcam), anti-Atg5 (1:1000, ab108327, Abcam), anti-Atg7 (1:10,000, ab133528, Abcam) and anti-GAPDH (1:1000, ab8245, Abcam) and exposed to HRP-linked anti-rabbit IgG secondary antibody (1:5000, Abcam). The relative density of each band was determined employing Image J software. Then the relative protein expression levels were presented as the ratio of the density values of bands between experimental and control samples.

### In vitro HUVEC tube formation assay

The MDA-MB-436 cell culture medium was changed to serum-free DMEM medium for 48 h and then collected, centrifuged and filtered to obtain tumor-conditioned medium (TCM). To solidify the gel, 300 μL of growth factor-reduced Matrigel (BD Biosciences, USA) was added into precooled 48-well plates and incubated for 30–60 min at 37 °C. Subsequently, human umbilical vein endothelial cells (HUVECs, 2 × 10^4^) were seeded on the gel with 200 μl of TCM concentrated 75-fold using an ultrafiltration device (Millipore, USA). The TCM from MCF-7 cells was supplemented with 1% FBS. The tube-forming ability of cells was calculated applying an inverted light microscope.

### Cell transfection

The overexpression plasmids of OPN (OPN-OE#1 and OPN-OE#2) and the corresponding control (vector) were synthesized by GenePharma Co., Ltd. (Shanghai, China). After that, the transfection of these vectors into cells was conducted adopting Lipofectamine 3000 reagent (Beijing Ya'anda Biotechnology Co., Ltd.) in light of the recommended protocol.

### Mice xenograft models

A total of 23 nude mice were acquired and tumors were developed in mice at a success rate of around 90%. Nude mice were intramuscularly injected with MDA-MB-436 cells in the right hind thigh at a density of 2 × 10^6^ per ml. The transplanted nude mice were separated into four groups at random: control, vector, OPN-OE and OPN-OE + LY294002 (*n* = 5). OPN-OE overexpression plasmids were injected into the tail vein of mice in the OPN-OE and OPN-OE + LY294002 groups. Mice in the vector group were injected with blank control plasmids via the tail vein. Mice in the control group were left untreated. Mice in the OPN-OE + LY294002 group were injected with 10 µl LY294002 once every 4 days. All mice were examined every 3 days and killed 21 days after tumor inoculation. Tumor tissues were taken for subsequent detection of related indicators.

### Immunohistochemistry (IHC)

Slides with paraffin sections were deparaffinized and dehydrated and IHC analysis was performed. Anti-VWF (1:200, ab287962, Abcam) was used as primary antibody. HRP-conjugated secondary Ab served as secondary antibody. Photographs were captured employing an Olympus-IX71 microscope (magnification, × 400).

### Measurement of MDA and GSH contents in tumor tissues and cells

MDA and GSH were detected in the animals: the nude mice were killed after successful modeling and then the tumor tissues of the mice were removed to detect the expressions of MDA and GSH in tumor tissues. After the corresponding treatment, the cells were collected and the corresponding kits were used to detect the expressions of GSH and MDA in the cells. The enzymatic activities of MDA (Cat.no.A003-1–2) and GSH (Cat.no. A005-1-2) were assessed with different commercial assay kits (Nanjing Jiancheng Bioengineering Institute).

### Statistical analysis

All data were analyzed utilizing SPSS 20.0 software and displayed in the form of mean ± SD. Student’s *t* test and one-way ANOVA were applied for statistical comparisons of the two groups and multiple groups. *P* < 0.05 meant that the experimental figures exhibited statistical significance. The sample size of each experiment group was greater than or equal to 3.

## Results

### Tumor sphere formation and angiogenesis abilities of MCF-10A, MCF-7 and MDA-MB-436 cells

**Fig. 1:**
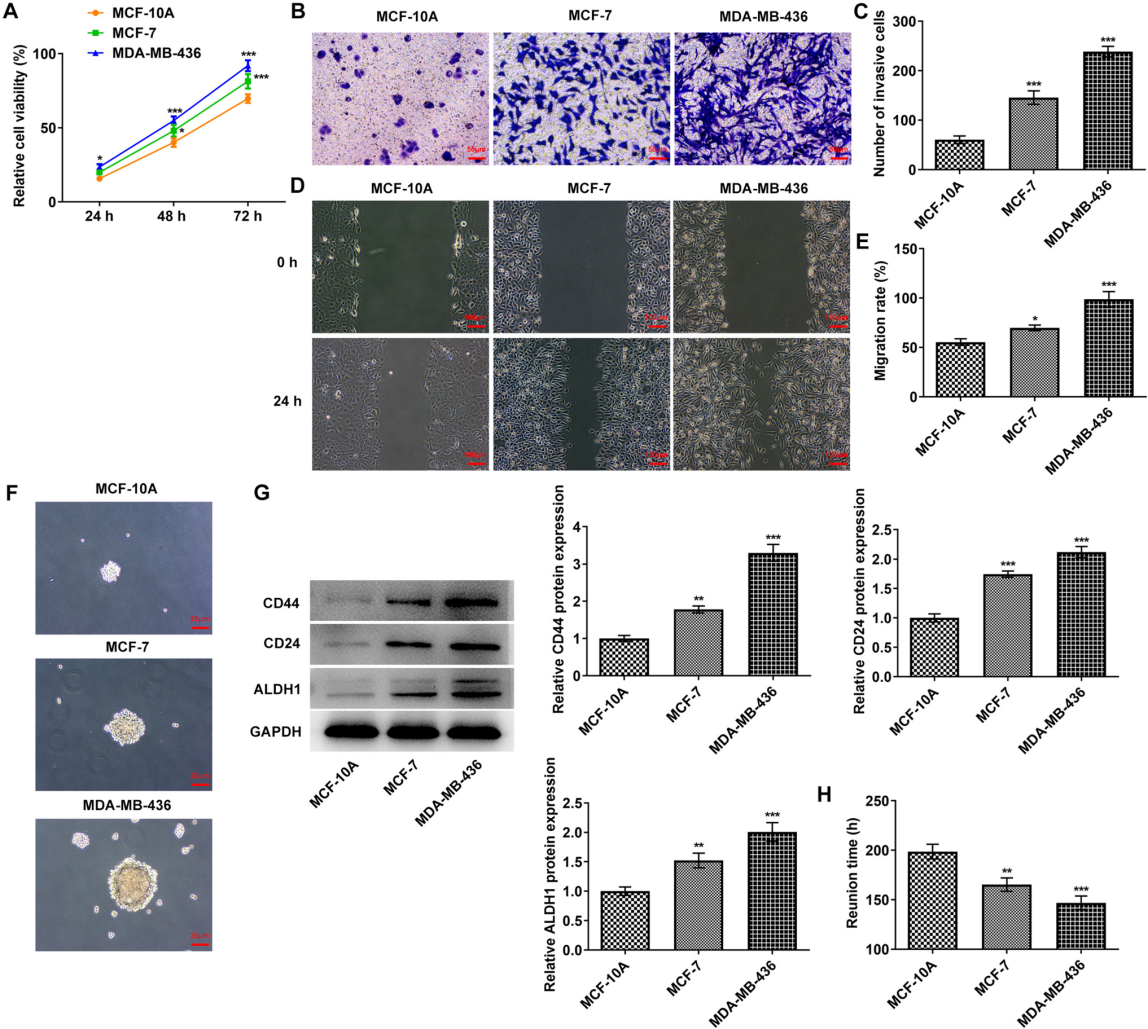
Tumor sphere formation of MCF-10A, MCF-7 and MDA-MB-436 cells. **A** A CCK8 kit was used to detect the cell proliferative capacity. **B** Transwell detected the cell invasion ability. **C** Statistical analysis of cell invasion ability. **D** Wound healing detected the cell migration ability. E. Statistical analysis of cell migration ability. **F** Tumor sphere formation of each group was detected by tumor sphere formation assay. **G** Western blot detected the expressions of CD44, CD24 and ALDH1. **H** Statistical analysis of the time of tumor sphere formation. *p < 0.05, **p < 0.01, ***p < 0.001 vs MCF-10A

**Fig. 2:**
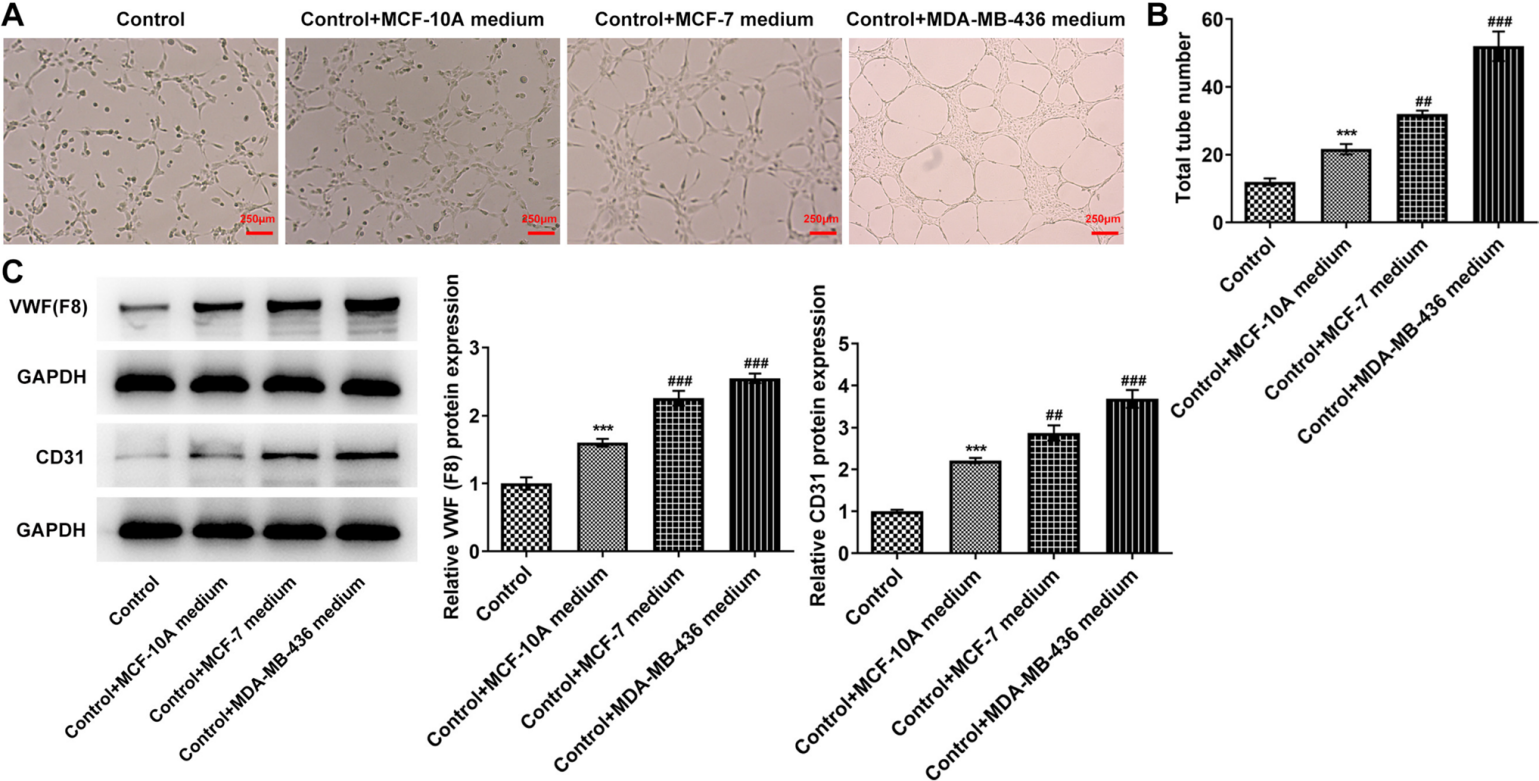
Cell angiogenesis abilities of MCF-10A, MCF-7 and MDA-MB-436 cells. **A** HUVEC tube formation assay was used to detect the angiogenesis abilities. **B** Statistical analysis of tube numbers. **C** Western blot detected the expressions of VWF and CD31. ***p < 0.001 vs control; ^##^*p* < 0.01, ^###^*p* < 0.001 vs control + MCF-7 medium

CCK8 was employed for the estimation of cell proliferative ability. Compared with MCF-10A cells, the proliferative ability of MDA-MB-436 and MCF-7 cells was rapidly enhanced (Fig. 1A). Transwell (Fig. 1B, C) as well as wound healing (Fig. 1D, E) was applied for the evaluation of cell invasive and migratory abilities, and it was discovered that in comparison to MCF-10A cells, the invasive and migratory abilities of MCF-7 and MDA-MB-436 cells were markedly elevated, particularly in MDA-MB-436 cells. Tumor sphere formation experiments showed that the tumor sphere formation ability of MDA-MB-436 cells was stronger than that of MCF-10A and MCF-7 cells (Fig. 1F). Western blot was adopted to assess the expression of cancer stem cell markers CD44, CD24 and ALDH1. The results in Fig. 1G exhibited that the contents of CD44, CD24 and ALDH1 in MDA-MB-436 and MCF-7 cells were remarkably increased. The reunion time for tumor sphere formation after disruption was also prominently reduced in MCF-7 and MDA-MB-436 cells by contrast with MCF-10A cells (Fig. 1H). Tubule formation assay detected the endothelial cell tubule formation abilities in tumor cells. The results showed a remarkable increase in tubular structure formation in HUVECs cultured with MDA-MB-436 medium compared with that in HUVECs cultured with MCF-10A medium (Fig. 2A, B). As Fig. 2C depicts, the levels of VWF and CD31 in MDA-MB-436 medium group were conspicuously enhanced when compared to the MCF-10A medium group.

### OPN regulates tumor sphere formation and vascular formation by activating the PI3K/AKT/mTOR signaling pathway in TNBC

**Fig. 3:**
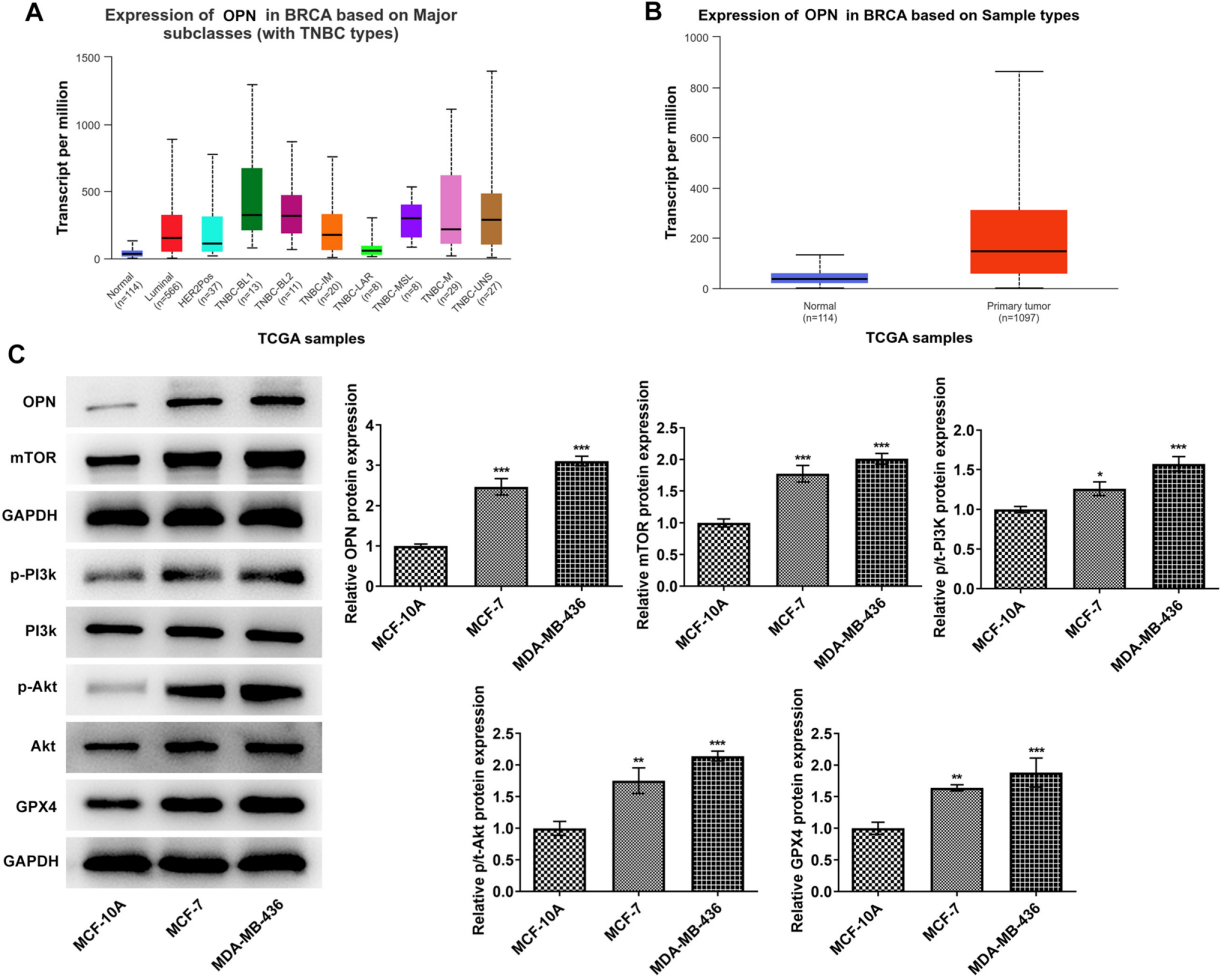
The expression of OPN and the PI3K/AKT/mTOR signaling pathway-related proteins. **A**, **B** UALCAN database-predicted OPN expression in breast cancer tissues. **C** Western blot detected the expressions of OPN and PI3K/AKT/mTOR signaling pathway-related proteins. *p < 0.05, **p < 0.01, ***p < 0.001 vs MCF-10A

**Fig. 4:**
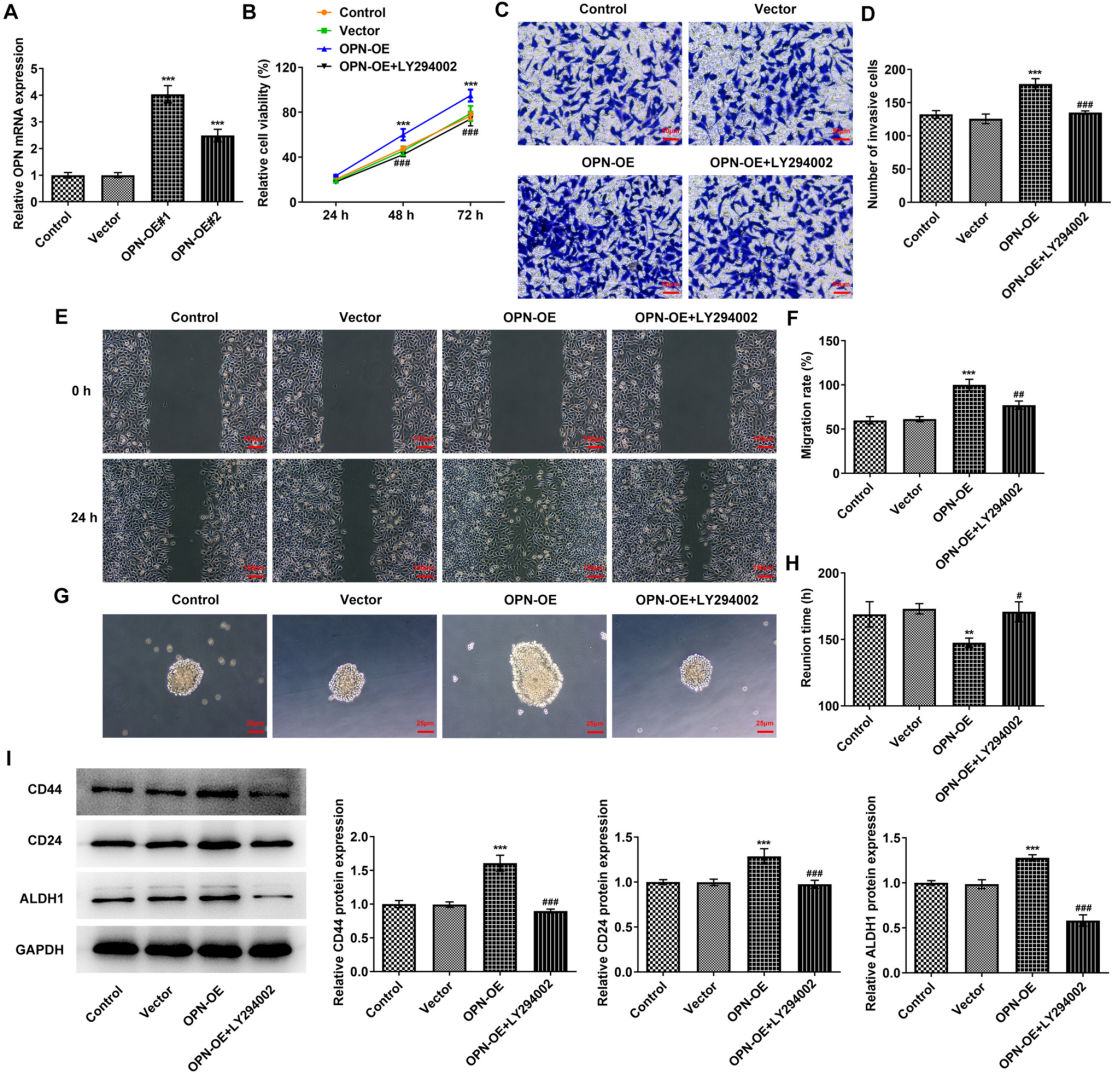
OPN regulates tumor sphere formation by activating the PI3K/AKT/mTOR signaling pathway in TNBC. **A** RT-qPCR detected the expression of OPN. **B** CCK8 kit was used to detect the cell proliferative capacity. **C** Transwell detected the cell invasion ability. **D** Statistical analysis of cell invasion ability. **E** Wound healing detected the cell migration ability. **F** Statistical analysis of cell migration ability. **G** Tumor sphere formation of each group was detected by tumor-sphere formation assay. **H** Statistical analysis of the time of tumor sphere formation. **I** Western blot detected the expressions of CD44, CD24 and ALDH1. **p < 0.01, ***p < 0.001 vs vector; ^#^*p* < 0.05, ^##^*p* < 0.01, ^###^*p* < 0.001 vs OPN-OE

**Fig. 5:**
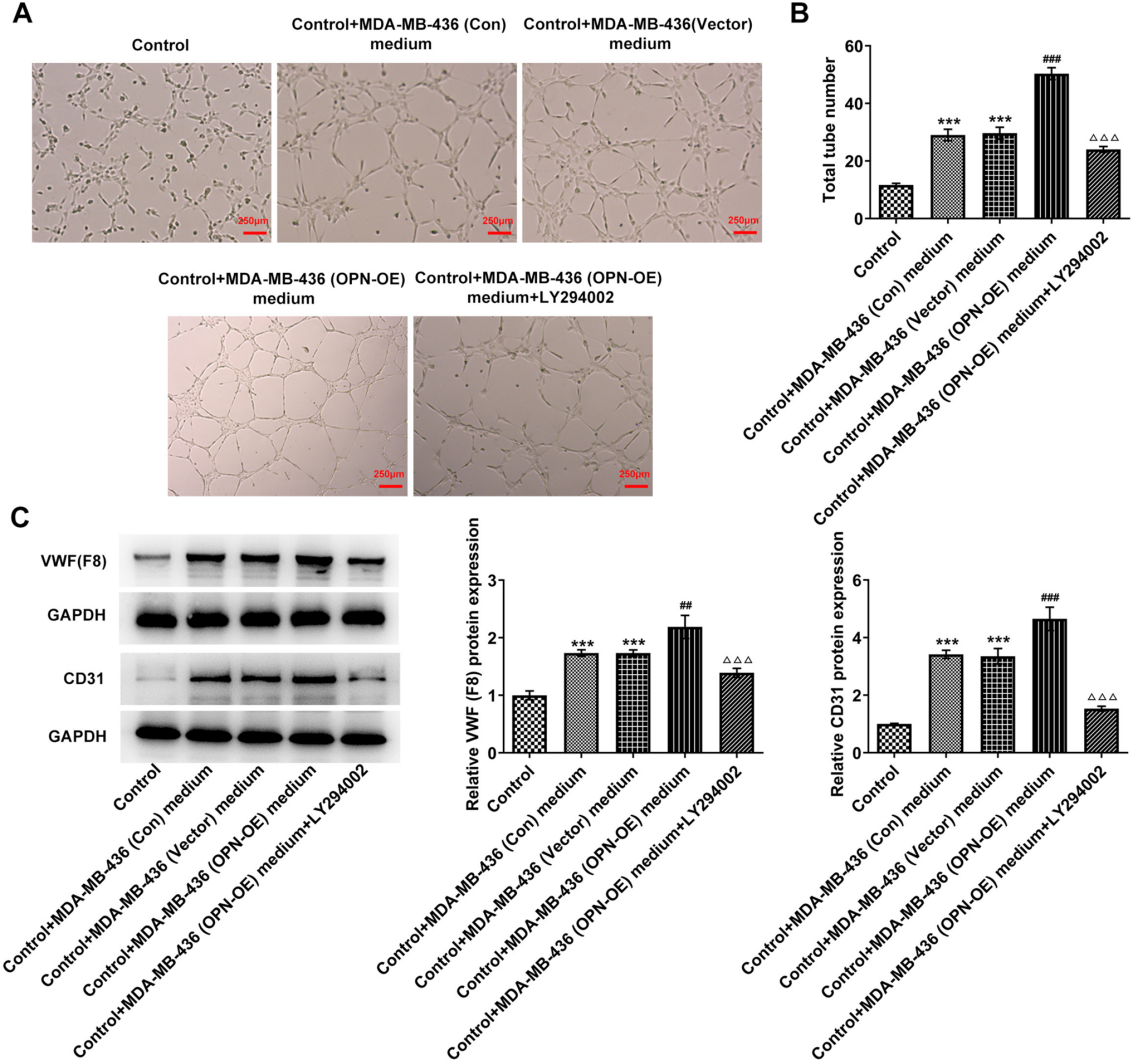
OPN regulates cell vascular formation by activating the PI3K/AKT/mTOR signaling pathway in TNBC. **A** HUVEC tube formation assay was used to detect the angiogenesis abilities. B Statistical analysis of tube numbers. C Western blot detected the expressions of VWF and CD31. ***p < 0.001 vs control; ^##^*p* < 0.01, ^###^*p* < 0.001 vs control + MDA-MB-436 (vector) medium; △△△*p* < 0.001 vs control + MDA-MB-436 (OPN-OE) medium

Based on the UALCAN database, OPN was predicted to be prominently overexpressed in breast cancer tissues (Fig. 3A, B). Subsequently, the contents of OPN and PI3K/AKT/mTOR pathway-related proteins were assessed and it was discovered that in comparison with MCF-10A cells, the contents of OPN, p-PI3K, p-Akt, mTOR as well as GPX4 were dramatically increased in MCF-7 and MDA-MB-436 cells (Fig. 3C). Then OPN overexpression vectors were constructed and transfected into MDA-MB-436 cells. RT-qPCR was used to detect the transfection efficiency (Fig. 4A). In addition, PI3K/AKT/mTOR signaling pathway inhibitor LY294002 was also added and cells were separated into the control, vector, OPN-OE and OPN-OE + LY294002 groups. Results from CCK8 displayed that the activity in the OPN-OE group was rapidly enhanced when compared to the vector group, while the activity in the OPN-OE + LY294002 group was reversed compared with the OPN-OE group (Fig. 4B). Transwell (Fig. 4C, D) and wound healing (Fig. 4E, F) experiments showed that the invasive and migratory abilities of the OPN-OE group were greatly increased compared with the vector group, and those of the OPN-OE + LY294002 group were tremendously reversed. Tumor sphere formation experiment results showed that the tumor sphere formation rate of MDA-MB-436 cells was increased after OPN was overexpressed, which subsequently declined to some extent following the inactivation of PI3K/AKT/mTOR signaling pathway (Fig. 4G, H). Results in Fig. 4I exhibit that the contents of CD44, CD24 and ALDH1 were elevated in MDA-MB-436 cells after OPN was overexpressed, which was reversed after blocking the PI3K/AKT/mTOR pathway. Tubule formation experiments as well as Western blot results demonstrated that in comparison with the vector group, tubular structure formation was markedly elevated in HUVECs in the OPN-OE group (Fig. 5A, B) and the contents of VWF and CD31 were rapidly elevated (Fig. 5C). In comparison with the OPN-OE group, the tubular structure formation of the HUVECs in the OPN-OE + LY294002 group was greatly diminished and the contents of VWF and CD31 were remarkably decreased.

### OPN regulates GPX4-mediated anti-lipid peroxidation by activating the PI3K/AKT/mTOR signaling pathway to affect tumor sphere formation and vascular formation in TNBC

**Fig. 6:**
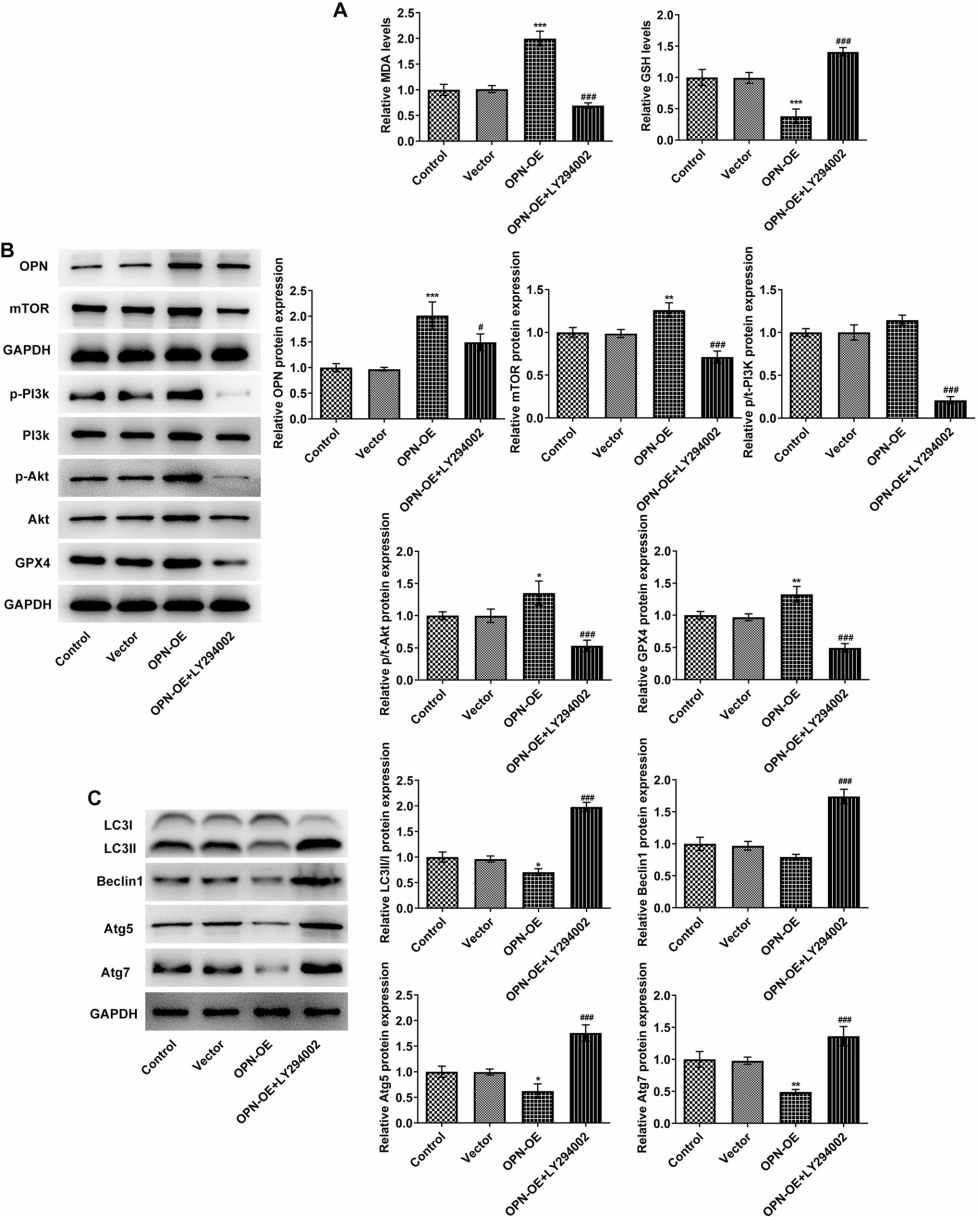
OPN regulates GPX4-mediated anti-lipid peroxidation by activating PI3K/AKT/mTOR signaling pathway to affect tumor sphere formation and vascular formation in TNBC. **A** The levels of MDA and GSD were detected with corresponding kits. **B** Western blot detected the expressions of OPN and PI3K/AKT/mTOR signaling pathway-related proteins. **C** Western blot detected the expressions of autophagy-related proteins in the downstream of mTOR signaling. *p < 0.05, **p < 0.01, ***p < 0.001 vs vector; ^#^*p* < 0.05, ^###^*p* < 0.001 vs OPN-OE

As Fig. 6A demonstrated, MDA activity in cells was conspicuously elevated, while GSH expression was cut down after OPN was overexpressed compared with the vector. However, after the administration of LY294002, MDA expression rapidly decreased and GSH expression increased in OPN + OE + LY294002 (Fig. 6A). Western blot was adopted to evaluate the expressions of OPN, PI3K/AKT/mTOR pathway- and autophagy-related proteins in the downstream of mTOR signaling. It was discovered that OPN, p-Akt, mTOR as well as GPX4 expressions were remarkably enhanced, while LC3II/I, Atg5 and Atg7 expressions were distinctly reduced in the OPN-OE group in comparison to the vector group. After further administration of LY294002, the contents of these proteins in cells were partially reversed significantly (Fig. 6B, C). The above findings implied that OPN could activate GPX4 expression, reduce the anti-lipid peroxidation level of cells and promote the development of TNBC by activating the PI3K/AKT/mTOR pathway.

### OPN regulates GPX4-mediated anti-lipid peroxidation by activating the PI3K/AKT/mTOR signaling pathway to affect tumor sphere formation and vascular formation in TNBC in vivo

**Fig. 7:**
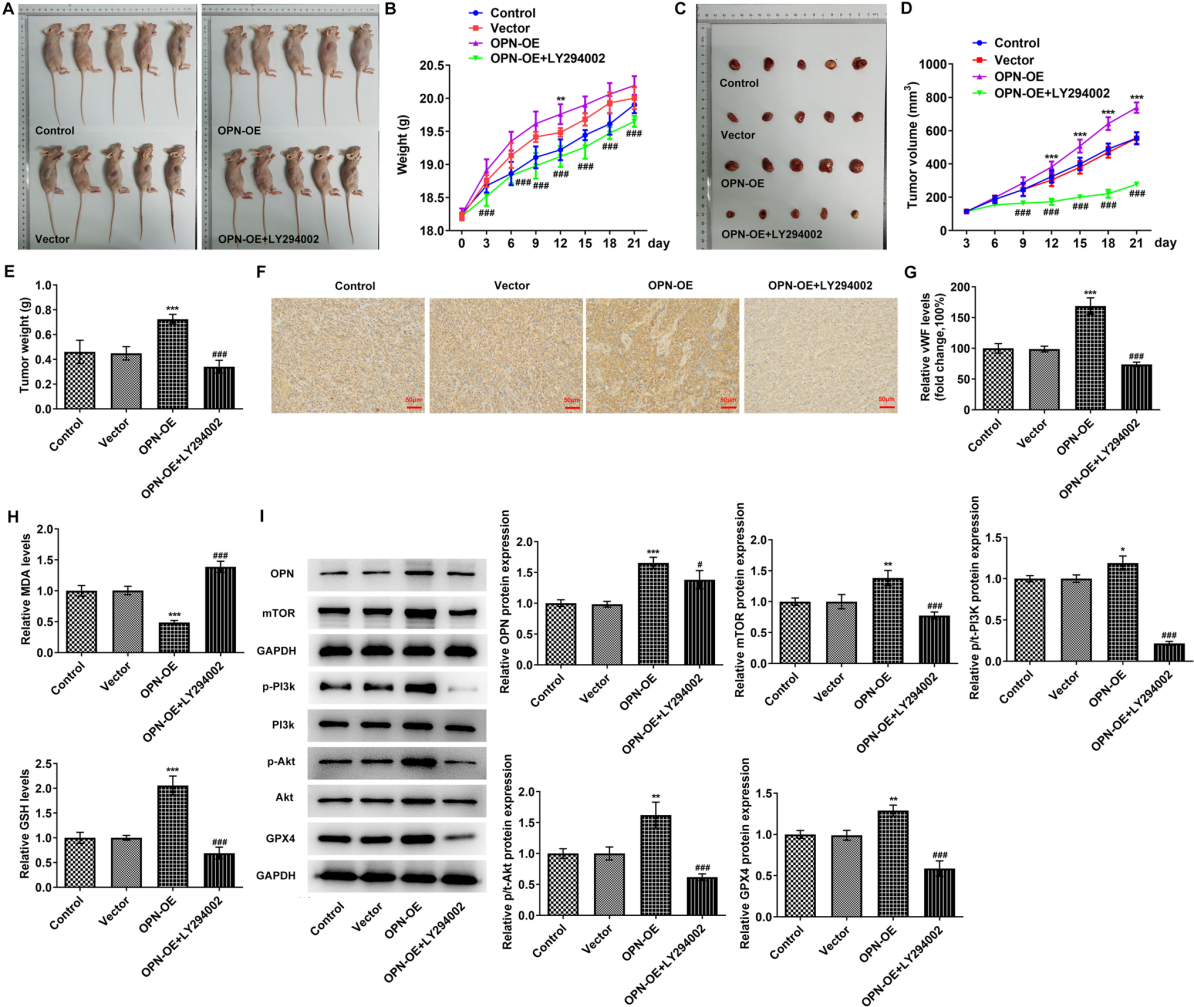
OPN regulates GPX4-mediated anti-lipid peroxidation by activating PI3K/AKT/mTOR signaling pathway to affect cell tumor sphere formation and vascular formation in TNBC in vivo. **A** The mice were photographed. **B** The weight of the mice. **C** The tumor of mice was photographed. **D** The tumor volume was observed. **E** The tumor weight was observed. **F**, **G** IHC detected the expression of VWF. **H** The levels of MDA and GSD were detected with corresponding kits. **I** Western blot detected the expressions of OPN and PI3K/AKT/mTOR signaling pathway-related proteins. *n* = 5. *p < 0.05, **p < 0.01, ***p < 0.001 vs vector; ^#^*p* < 0.05, ^###^*p* < 0.001 vs OPN-OE

Finally, the tumor cells were cultured and inoculated subcutaneously into mice; then the mice were photographed and the tumor size was observed. We found that body weight and tumor size in the OPN-OE group increased. In comparison with the OPN-OE group, body weight and tumor volume were conspicuously reduced in the OPN-OE + LY294002 group (Fig. 7A–E). Results obtained from IHC demonstrated that the VWF level was markedly enhanced in the OPN-OE group in comparison with the vector group. When compared to the OPN-OE group, the VWF level in the OPN-OE + LY294002 group rapidly decreased (Fig. 7F, G). The oxidative stress level in tumor tissues was shown in Fig. 7H. After OPN was overexpressed, the MDA activity in tumor tissues was dramatically cut down, while GSH activity was enhanced (Fig. 7H). It was also discovered that the contents of OPN, p-PI3K, p-Akt, mTOR as well as GPX4 in tumor tissues of mice in the OPN-OE group were rapidly elevated in comparison with the Vector group. In contrast with the OPN-OE group, the levels of OPN, p-PI3K, p-Akt, mTOR as well as GPX4 were greatly declined in the OPN-OE + LY294002 group (Fig. 7I).

## Discussion

TNBC, which accounts for approximately 15% of all breast cancers, differs from other traditional breast cancers (Ali et al. 2017). Studies have evidenced that in comparison with non-TNBC patients, TNBC patients have worse survival (Li et al. 2017), higher clinicopathological characteristics (Kim et al. 2016) and exhibit more aggressive pathology (Agarwal et al. 2016). In our experiment, it was found that the proliferation capacity of TNBC cell line MDA-MB-436 was greatly enhanced in comparison with non-TNBC cell line MCF-7 and normal cell line MCF-10A. In addition, it was displayed that TNBC cells had stronger invasive and migratory abilities. In addition, the tumor sphere formation and angiogenesis abilities of MDA-MB-436 cells were also higher than that of MCF-7 and MCF-10A cells.

OPN is located on chromosome 4Q24-Q25 and consists of seven exons and six introns (Sørensen and Christensen 2023). It is expressed in a variety of human tissues (Sørensen and Christensen 2023). OPN contains specific arG-GLy-ASP sequence related to cell adhesion, which promotes cell chemotaxis, adhesion and migration by binding to its receptor integrin, etc. (Subraman, et al. 2015), mediating the occurrence and development of tumors (Chiodoni et al. 2015). In our experiment, the location of the OPN PCR promoter sequence was consistent with it. Multiple studies in human samples and tumor-bearing mice have shown that as a multifunctional protein, OPN expression is increased and has close relation with the occurrence, development and metastasis of laryngeal squamous cell carcinoma (Chen et al. 2015), diffuse large B-cell lymphoma (He et al. 2023), hepatocellular carcinoma (Zhu et al. 2018), etc. Notably, a previous study has shown that elevated OPN expression contributes to more aggressive behaviors of breast cancer cells and can be used as a diagnostic and prognostic marker of breast cancer (Thorat et al. 2013). In 115 patients suffering from breast cancer, OPN expression was markedly enhanced, and the high expression of OPN was closely related to adverse reactions and overall survival rate (Elbaiomy et al. 2020). Of note, OPN has also been noted to display upregulated expression in HER2-positive and triple-negative/basal-like tumors (Ortiz-Martínez et al. 2014), suggesting the possible role of OPN as an oncogene in TNBC. In our experiment, bioinformatics tools predicted that OPN expression was upregulated in breast cancer tissues and it was also uncovered that the OPN level in MCF-7 and MDA-MB-436 cells was conspicuously elevated.

Angiogenesis has a decisive role in the growth of aggressive tumors. Tumor angiogenesis is divided into endothelial cell activation, proliferation, migration, production of vascular growth factors in tumors and surrounding tissues as well as tissue infiltration of endothelial cells (Viallard and Larrivee 2017). Therefore, the study of dysangiogenesis in tumors helps to develop new anti-angiogenesis therapies. OPN can promote vascular endothelial growth factor (VEGF) secretion in breast cancer cells (Chakraborty et al. 2008) and prostate cancer cells (Wisniewski et al. 2019), and affect vascular formation through VEGF. These results indicate that OPN plays a role in inducing new angiogenesis. In this paper, we noticed that overexpression of OPN in MDA-MB-436 cells could further increase the proliferative, invasive, migratory, tumor sphere formation and angiogenesis abilities of MDA-MB-436 cells, confirming the potential oncogenic role of OPN in TNBC for the first time.

The PI3K/Akt/mTOR pathway acts as a critical player in tumor cell proliferation, vascular growth as well as metastasis (Glaviano et al. 2023). Also, downstream mTOR is a vital protein kinase of this pathway able to regulate the biological effects of tumor cell growth, proliferative ability, survival as well as angiogenesis (Tian et al. 2019). It is shown that the PI3K/Akt/mTOR pathway is triggered in TNBC, and PI3K/Akt/mTOR pathway inhibitors can inhibit the tumor growth of TNBC and cause the apoptosis of cancer cells conversely (Costa et al. 2018; Massihnia et al. 2016). Actin-like protein 8 promotes the proliferative, migratory and invasive abilities of TNBC cells and inhibits apoptosis by triggering the PI3K/AKT/mTOR pathway (Fan et al. 2021). Notably, OPN can induce proliferation, invasion, migration and angiogenesis in tumor cells by activating the PI3K/AKT/mTOR pathway (Santos et al. 2022). In our experiment, it was found that the PI3K/AKT/mTOR signaling was activated in MDA-MB-436 cells compared with normal cells and non-TNBC cells. Inhibition of PI3K/AKT/mTOR signaling could significantly reverse the effects of OPN overexpression on cell malignant progression. These findings implied that OPN in TNBC cells drove the advancement of TNBC by triggering the PI3K/AKT/mTOR signaling pathway.

Ferroptosis is a form of cell death due to missed control of membrane lipid peroxidation (Ursini and Maiorino 2020). Accumulating evidence has highlighted the protective role of ferroptosis activation in TNBC (Xu et al. 2022; Li et al. 2023). GPXs are a class of enzymes responsible for the reduction of hydroperoxide in a glutathione-consuming manner and thus regulate cellular redox homeostasis (Schwarz et al. 2023). Among them, GPX4, located on various membrane structures in the cytoplasm, has emerged as a lipid repair enzyme, which protects lipid structures on various membranes in cells from oxidative stress damage and maintains the integrity of membrane structures and normal organelles by catalyzing the reduction of lipid peroxides, and thus serving as a negative key regulator of ferroptosis (Liu et al. 2023). GPX4 is widely expressed in many cancers and acts as a critical oncogenic gene (Xia et al. 2019; Wang, et al. 2021; Kim et al. 2020). Increasing researches have proposed that GPX4-mediated anti-lipid peroxidation plays an important role in TNBC (Ding et al. 2021; Chen et al. 2023). A case of previous study has found that mTOR is correlated with GPX4 expression and the interaction between the two can regulate autophagy-dependent cancer cell death (Liu et al. 2021). For instance, GPX4 degradation and mTOR inhibition mediated by autophagy play a synergistic role in killing bladder cancer cells (Sun et al. 2021). Therefore, in the current work, it was speculated that OPN regulated GPX4-mediated anti-lipid peroxidation by regulating the PI3K/AKT/mTOR signaling pathway. Concurrently, our study uncovered that GPX4 expression was greatly increased in MDA-MB-436 cells. OPN overexpression could promote GPX4 level and decrease autophagy-related LC3II/I, Atg5 and Atg7 expressions in tumor cells, which was subsequently reversed by LY294002 administration. This finding further proposed that OPN might activate PI3K/AKT/mTOR signaling to increase GPX4 expression to suppress lipid peroxidation and subsequent ferroptosis, eventually contributing to carcinogenesis and tumor metastasis in TNBC.

We will further explore the effects of inhibiting OPN expression in breast cancer cells on tumor sphere formation and angiogenesis in future experiments. In addition, we will use LY294002 alone to further verify the effects of LY294002 alone on tumor sphere formation and angiogenesis. Besides, emerging biomarkers have been mentioned to play important roles in TNBC, such as TRPS1 (Ai et al. 2021), LAG-3 (Tahtacı et al. 2023), KLK4 (Yang et al. 2017) and so on, the detailed functional mechanisms of which also need to be figured out. Meanwhile, whether OPN may interact with one of the potential biomarkers to function in TNBC also demands investigation.

## Conclusion

In conclusion, we found that OPN could promote tumor sphere formation and angiogenesis in TNBC by activating the PI3K/AKT/mTOR pathway to regulate GPX4-mediated anti-lipid peroxidation levels.

## Data Availability

The analyzed datasets generated during the present study are available from the corresponding author on reasonable request.
